# Identifying Work-Related Internet’s Uses—at Work and Outside Usual Workplaces and Hours—and Their Relationships With Work–Home Interface, Work Engagement, and Problematic Internet Behavior

**DOI:** 10.3389/fpsyg.2019.02118

**Published:** 2019-10-11

**Authors:** Emilie Vayre, Anne-Marie Vonthron

**Affiliations:** Parisian Laboratory of Social Psychology (LAPPS, EA 4386)—Work, Ergonomic, Guidance and Organizations Research Group (TE2O), Department of Psychology, Université Paris Nanterre, Nanterre, France

**Keywords:** work-related internet’s uses, intensity–places–time periods of internet uses for work, work–home interface, work engagement, problematic Internet use

## Abstract

Many studies have analyzed the uses of information and communication technologies (ICTs) for work, with some focusing on use at the office and others on use outside the traditional workplace and workday. However, there is little research encompassing all work uses of ICTs, both in and out of the office, and on the ways in which they affect employees’ attitudes toward their work and quality of life. Thus, the present study aims to (a) explore the links between intensity, places, and time periods of using the Internet for work; (b) examine whether Internet uses for work are related to the perceived impact of work on personal life, work engagement, and Internet addiction. An empirical study was conducted based on a questionnaire survey of 502 executives. We measured their use of the Internet for business purposes both in and outside of the standard workday/workplace; the perceived impact of work on their personal life; their work engagement; and their relationship to the Internet. Four categories of Internet use for work were identified (Cluster analysis). They differed with respect to intensity, places, and time periods dedicated to Internet uses (at standard workplace, at home, while traveling; during a typical workday, a day off, or vacation). The results obtained from Multinomial Logistic Regression show that technological devices provided by the employer and personal uses of the Internet are related to the intensity, places, and time periods of executives’ work-related Internet uses. Furthermore, ANCOVAs reveal that high-intensive, extensive, and porous Internet uses for work appear to foster the permeability between work and personal life, diminish managers’ dedication and vigor at work, and favor Internet addiction. Based on these findings, we discuss the importance of the “right to disconnect” and prevention programs regarding Internet uses, two major issues that attract the attention of organizations as well as public health authorities.

## Introduction

Advanced information and communication technologies (ICTs) have become increasingly prevalent in companies and are revolutionizing work habits. Because ICTs enable employees to work anywhere, they may also be expected to work anytime, meaning that there is no longer any boundary between work time and personal time (e.g., de Wet and Koekemoer, 2016; Carlson et al., 2018). Indeed, being constantly connected has led to an increase in working hours, extending them outside the traditional workday (e.g., Boswell and Olson-Buchanan, 2007; Senarathne Tennakoon et al., 2013; Thomas, 2014). Henceforth, a growing number of employees work both at home and at their company’s offices *via* ICTs, to such an extent that some researchers find that work is now being “offshored” into the domestic sphere (Halford, 2005). This reconfiguration of work times and places has consequences on work behaviors, individuals’ relationships to work, and the boundary between work and personal life.

The central purpose of the current study is, first, to explore work-related Internet uses, both in and outside traditional workplaces and hours. The majority of previous studies have examined either the intensity of ICTs use, the use during a single period (at work or outside working hours), or the use in a specific location (at work or at home), but seldom all the three. Secondly, the aim of our research is to identify to what extent and in what way different categories of Internet uses for work are related to work–home interaction, work engagement, and the relationship to the Internet. As we shall see, even though the phenomenon of work spilling over into personal life and the use of ICTs for work is a central topic of research, there is no consensus on how ICTs uses affect work–non-work interference (WNWI). Moreover, little empirical research has been done to explain the links between work-related ICTs uses and work engagement or the relationship to technology. Thus, this study seeks to contribute to and deepen the knowledge in the literature of this field. Finally, a complementary goal of this study is to identify and control the potential role of socio-demographic and situational characteristics both on the Internet uses for professional purposes and on the outcome variables considered.

In this perspective, we conducted an empirical study among 502 managerial professionals, which enabled us to refine our understanding of the links between the variables considered. According to the literature presented below, we expected that work–home interaction, work engagement, and the relationship to the Internet differed with respect to intensity, places, and time periods dedicated to Internet uses. That is, the more Internet uses are intensive, extended, and porous, the higher the levels of WNWI, the lower the levels of work engagement and the higher the level of problematic relationship to the Internet are.

In practice, this study questions current organizational contexts and provides knowledge for human resource managers so that they may create an organizational climate and policies for the using the Internet for work and for work–life balance that promote employees’ well-being and quality of life as well as their work engagement.

### Work-Related ICTs Uses

The literature on the effects of ICTs use in the workplace reveals contradictory results. Some studies found that these technologies improve operations management, decision-making, and communication within organizations, and that they change working environments and management styles (Bobillier-Chaumon, 2003; Isaac et al., 2007). ICTs may also offer new ways to organize work and may increase employees’ autonomy and flexibility, allowing them to break free from the limits of the traditional workplace and workday (Bobillier-Chaumon, 2003; Colombier et al., 2007; Lee and Sawyer, 2010; Tremblay, 2012; Thomas, 2014).

Nevertheless, several studies have shown that more and more employees complain of an increase in workload, a longer workday, and feeling overloaded and overwhelmed (Thomas, 2014; Ninaus et al., 2015). While these new forms of work give employees greater flexibility, many complain of difficulty disengaging from work and report working longer, in the evening or on weekends at home (Fenner and Renn, 2010). Several authors consider that technologies facilitate the extension of work into personal life, by allowing employees to remain connected and continue working during non-work hours (Rey and Sitnikoff, 2004; Boswell and Olson-Buchanan, 2007; Senarathne Tennakoon et al., 2013). Therefore, these technologies are sometimes perceived as a way for employers to force employees to continue working after official hours (Wright et al., 2014). Indeed, current social norms and organizational culture, management, and evaluation systems encourage implicitly or explicitly the extension of work outside the usual times and places *via* ICTs (Clark, 2001; Eagly and Carli, 2007; Senarathne Tennakoon et al., 2013). Pressure from colleagues and superiors, as well as organizational demands, are factors that promote longer hours at the workplace and the feeling that one has to be permanently connected to work, to be accessible and responsive, in order to appear committed and productive (Fenner and Renn, 2010; Matusik and Mickel, 2011; Thomas, 2014; de Wet and Koekemoer, 2016).

In terms of consequences, numerous studies have shown that the increase in ICTs uses and ICT demands at work have deleterious effects on physical and mental health (fatigue, stress, feeling overwhelmed or lack of time, burnout; Ninaus et al., 2015; Stadin et al., 2016). However, as discussed in the next section, most studies focus on the deleterious effects of work intruding on the work–home interaction.

### Work-Related Uses of ICTs and Work–Life Interface

According to Geurts et al. (2005), work–home interaction refers to the process whereby an individual’s behavior in one area (e.g., the way he or she behaves at home) is influenced (negatively or positively) by reactions from another area (e.g., work). The relationship between these two domains of life has generated a great deal of research. Studies have clearly highlighted the deleterious consequences for the employee and for the work organization of the perception of a conflict between work and family life (e.g., Allen et al., 2000; Anderson et al., 2002; Major et al., 2002; Frone, 2003; Montgomery et al., 2003; Demerouti et al., 2004).

Several empirical studies reveal that the growth of technology and its uses increase the fragmenting of work tasks and the workday, blur the boundary between work and personal life, and encourage the invasion of professional life into personal life (Genin, 2009; Tabassum and Rahman, 2013; de Wet and Koekemoer, 2016; Kossek, 2016). Indeed, the use of ICTs for work fosters the encroachment of work on life outside of work, and makes the border between personal life and work life more porous, as employees find it very difficult to establish a limit between these two areas of existence (Isaac et al., 2007; Park and Jex, 2011; Thomas, 2014; de Wet and Koekemoer, 2016).

With regard to the use of ICT outside traditional workplaces and hours, studies show that the intrusion of work into personal life is often the cause of conflicts with partners, family members, and more broadly significant others (Kossek and Lautsch, 2008; Leonardi et al., 2010; Ayyagari et al., 2011). Many researchers found that employees increasingly respond, or are encouraged to respond, to e-mails, text messages, or professional phone calls during their free time, which leads to work overload, role ambiguity, and work–life conflict, ultimately promoting the perception of stress, technostress, techno-invasion, and burnout (Isaac et al., 2007; Kossek and Lautsch, 2008; Genin, 2009; Leonardi et al., 2010; Ayyagari et al., 2011; Park and Jex, 2011; Tabassum and Rahman, 2013; Thomas, 2014; Ferguson et al., 2016; Leung and Zhang, 2017). Previous studies generally point out that the use of ICTs for professional purposes outside usual working hours favors disruption of family activities and the perception of work–family conflict, from the point of view of employees as well as their significant others (Boswell and Olson-Buchanan, 2007; Fenner and Renn, 2010; Matusik and Mickel, 2011; Wright et al., 2014; Carlson et al., 2018). Although employees are aware of the negative implications of extending work *via* ICTs, they give in to the pressure to stay connected to their job (Waller and Ragsdell, 2012).

However, some studies, even though they are rare, are more nuanced and emphasize that while technology leads to an intensification of work and an invasion of the personal sphere, at the same time, they allow people to better reconcile them with personal demands, and to more effectively control the boundary between work and non-work (Wajcman et al., 2008; Land and Taylor, 2010; Chesley, 2014; Ninaus et al., 2015). Batt and Valcour (2003) and Ninaus et al. (2015) found that ICTs blur the distinction between these two areas of life and increase the perception of work–family conflict, while also demonstrating that ICTs allowed for a better balance between work and private life, a greater flexibility, and the opportunity to better manage job demands. In the same vein, the results of Ghislieri et al. (2017) indicated that off-work hours technology-assisted job demand was positively related to work–life conflict and to work–life enrichment (but only in the male group). For their part, Derks et al. (2016) showed that for employees who prefer work and family roles to be integrated, the smartphone has the potential to reduce work–family conflict and enhance family role performance.

Hence, it can be assumed that the more intensive, extensive, and porous the use of the Internet for professional purposes is, the more employees perceive a negative impact of work on non-work (H1a), and at the same time, the more they perceive a positive interference from work with home (H1b). Stated differently, we posit that using the Internet for work, both at work and outside traditional workplaces and hours, fosters the permeability between work life and personal life.

### Professional Uses of ICTs and Work Engagement

Although there are several conceptual frameworks for defining work engagement, Eldor (2016)’s literature review explains that most recent studies on the subject have adopted Schaufeli et al.’s perspective. In this approach, work engagement is characterized by a high level of energy, the ability to deal well with job demands, an effective connection with work activities, and strong identification with one’s work (Schaufeli et al., 2002, 2006; Bakker et al., 2008). Schaufeli et al. identified three components of work engagement. Vigor refers to a high level of energy and resilience at work as well as a willingness to make an effort and persist in the face of difficulties encountered in the workplace. Dedication involves a high level of investment, enthusiasm, and intellectual stimulation at work, of being inspired by and proud of one’s work, and feeling that it is meaningful work. Finally, absorption is defined as being completely focused on and captivated by one’s work so that one does not realize time passing and that it is hard to detach oneself from work.

Numerous studies have shown that work engagement promotes optimism, personal initiative, loyalty, positive attitudes, the implementation of proactive, and organizational citizenship behaviors, performance and employee retention, as well as the efficiency and success of work organizations (Bakker et al., 2006; Christian et al., 2011; Bhuvanaiah and Raya, 2016; Eldor, 2016). Research also showed that engagement and burnout are negatively correlated: vigor counters exhaustion and dedication opposes cynicism (Schaufeli et al., 2002, 2006).

To our knowledge, there is very little research that seeks to understand how using ICTs for professional purposes, whether at work or outside traditional workplaces and hours, affects employee engagement. Salanova and Llorens (2009) observed that the frequency of ICTs use for work is significantly and negatively correlated with work engagement (i.e., vigor, dedication, and absorption). Furthermore, the results of Ter Hoeven et al. (2016) reveal that ICTs use enhances work engagement if this use is associated with greater efficiency and accessibility in work communication and processes. However, when technology use fosters interruptions, it contributes to an increase in employees’ work-related burnout and a decrease in work engagement. Finally, the study of Boswell and Olson-Buchanan (2007) shows that the use of CTs outside working hours encourages job involvement (i.e., absorption in and importance of work). Nevertheless, the findings indicate that these uses are associated with less affective commitment to the company. The authors explain these contrasting results by the fact that these uses of CTs, which reflect significant investment in the working world, may be linked with a feeling of frustration or even burnout, which affects affective commitment. Ferguson et al. (2016)’ study, showing that the frequency of using mobile devices for work during family time is related to lower organizational commitment and greater burnout, reinforces this interpretation.

Therefore, we suspect that the more intensive, extensive, and porous the use of technologies and the Internet for work is, the less employees are engaged at work (i.e., low vigor, dedication, and absorption) (H2).

### Work-Related Use of ICTs and Problematic Relationship to the Internet

Although many terms are used, when referring to addiction or inappropriate or excessive uses of technologies, they refer mainly to the Internet (e.g., *Internet addiction*, Young, 1998; *problematic Internet use*, Caplan, 2002; *excessive Internet use*, Hansen, 2002; etc.). Whatever the definitions, the concept of Internet addiction implies a loss of behavioral control despite attempts to moderate or suspend using it (Young, 1998; Griffiths, 2003; Kim and Byrne, 2011). Studies in this field have shown that excessive use of the Internet can lead to neglecting work, domestic, and family responsibilities, and disrupt social relations, with users gradually shutting down to family relations and professional relationships (Widyanto and McMurran, 2004; Durand et al., 2008).

Even though the general duration of Internet use is not a criterion for Internet addiction, the length of time is a warning signal, as several studies show a positive and significant link between Internet addiction and the number of hours per week or per day of Internet use, regardless of the activities done or the applications used (Nalwa and Anand, 2003; Khazaal et al., 2008; Vanea, 2011). Problematic behavior studies in other domains have already shown that easier access leads to more frequent and regular use, which can lead to dependency, and that mobile devices investment is associated with Internet addiction (Griffiths, 2003; Harwood et al., 2014). Given that these technologies have become widespread, generalized, and part of most work environments, and that the workplace and tasks involve greater Internet access, the time spent on the Internet has increased.

However, there are little empirical data on problematic Internet use at work. Durand et al. (2008) found that certain work characteristics, such as high-performance goals and excessive responsibilities, lead to stress, whose symptoms may be coupled with behavioral addiction. In addition, Beard (2005) emphasized that cultural factors such as belonging to a technologically advanced society or the need to use the Internet for work encouraged Internet use to an extent that may be detrimental. More recently, the research of Salanova et al. (2013) showed that job demands in terms of work overload, role ambiguity, and mobbing, and lack of personal resources, in terms of emotional competence, positively predict technoaddiction among intensive technology users.

As communication technologies become increasingly important devices to workers, many people start to use them continuously to stay in constant contact with work. They become preoccupied with mobile activities and check uncontrollably their smartphone and electronic devices, which can lead to perceiving a work–life conflict, to experience anxiety, and ultimately to increased stress and even burnout (Durand et al., 2008; Wright et al., 2014; Li and Lin, 2019). In line with these observations, some studies have demonstrated that for workers who are severely dependent on smartphones at work, it is very hard for them to psychologically detach from their work and their phones, leading to serious anxiety and stress (Perlow, 2012; Derks and Bakker, 2014). Li and Lin (2019) also found that, the more employees depend on their smartphones for meeting work goals, the more they have various smartphone addiction symptoms, such as withdrawal and silence.

We thus hypothesize that the more intensive, extensive, and porous the use of technologies and the Internet for work is, the more workers engage in Internet behaviors that tended to be excessive (H3).

## Materials and Methods

### Participants and Procedure

Given our research objectives, we conduct a study among employees placed in executive positions, including managers who supervise a team and technical experts (Le Douarin, 2007). Indeed, ICT users at work are employees with higher degrees, in middle management or executive jobs (Colombier et al., 2007). Moreover, the increase in work hours associated with the growth of ICTs as well as the use of communication technologies outside working hours particularly affects managers (Boswell and Olson-Buchanan, 2007; Thomas, 2014).

We focused more specifically on executives working in or near a great metropolis. Similarly, we centered our research on employees not in teleworking (i.e., don’t work formally or contractually in teleworking).

To recruit executives, we contacted employees from our personal and professional networks^1^. Furthermore, we visited business districts with a high concentration of companies with these categories of employees. Questionnaire packages were distributed to participants. They had to return the filled-out questionnaires in a sealed envelope or directly by mail.

From the 750 distributed questionnaires, 502 questionnaires correctly completed were returned.

As indicated in Table 1, our sample is made up of almost as many women as men, aged 40 on average. We note that the executives questioned have one child on average and that most of them (72.7%) live in a couple or in a couple with their children. A majority of respondents hold positions of CEO, senior executive, or executive officer and work in the private sector.

**TABLE 1:** Participants’ socio-professional and socio-demographic characteristics.

		**M (σ)**	**Min–Max**
Portable connecting tools provided for by the employer (PCT-PE)		1.35 (0.82)	0–3
Personal Internet Use (PIU)—Workday		0.85 (0.95)	0.00–3.00
Personal Internet Use (PIU)—Day off		1.45 (1.20)	0.00–5.00
Personal Internet Use (PIU)—Day of leave		1.14 (1.28)	0.00–5.00
Age		39.98 (9.85)	22–67
Children		1.22 (1.17)	0–5

		***N***	**%**

Socio-professional category (SPC)	CEO, senior executive, executive officer	289	57.6
	Middle manager	213	42.4
Work sector	Private business	345	68.7
	Government employee or similar	157	31.3
Gender	Male	260	51.8
	Female	242	48.2
Household	Alone	72	14.3
composition	With companion	106	21.1
	With companion and children	259	51.6
	Alone with children/Shared house	65	13

### Measures

#### Socio-Professional and Socio-Demographic Characteristics

To identify the role of socio-demographic and socio-professional characteristics on work-related Internet uses, and on outcome variables (i.e., work/private life interference, work engagement, and problematic relationship to the Internet), we gathered some general information about the executives’ work-related and individual characteristics.

Participants were asked which technologies were used for work (nature and number) and whether they were provided by the employer. Time spent per day on the Internet for personal purposes was also evaluated (i.e., hours and minutes spent on the Internet during a typical working day, a typical weekly day off, and a typical day of vacation).

In addition, respondents also answered questions related to their socio-professional category, their work sector (private vs. public), their gender and their age, the number of their children, and the household composition.

#### Daily Internet Uses for Professional Purposes

To apprehend professional uses of Internet, we asked participants to report the time spent (hours and minutes) per day on the Internet as part of their job, depending on the following: (a) three distinct periods: during a typical working day, a typical weekly day off, and a typical day of vacation; and (b) three distinct locations (associated to each of the three periods): at work or at a place usually dedicated to work, at home, or while traveling on public transport (train, metro, bus, etc.). Concerning the typical working day, a distinction was made between the uses performed during standard work hours and at the standard workplace, and the uses realized outside working hours and places (in the early morning or evening, before or after work, at home or traveling).

In the questionnaire, it was indicated that these uses could be of different nature (i.e., sending work e-mails and documents, searching for information, visioconferences with co-workers or clients, etc.). In order to facilitate the reading of the results, during data entry, the usage times (hours and minutes) were rounded to the nearest quarter of an hour.

#### Work–Home Interference

The quality of the influence of work on private life was measured using the Survey work–home interaction—Nijmegen (Wagena and Geurts, 2000) in its adapted and validated French version (Lourel et al., 2005). This instrument captures four types of work/home interference: negative/positive interference from “work” with “home” and negative/positive interference from “home” with “work.”

Given the aims of our research (identify the consequences of Internet use for work), we focused solely on the first two dimensions. The negative impact of work on “non-work” (negative WNWI) was evaluated by eight items (e.g., “You do not fully enjoy the company of your spouse/family/friends because you worry about your work”). Similarly, the positive impact of work on private life (positive WNWI) refers to five items (e.g., “After a pleasant working day/working week, you feel more in the mood to engage in activities with your spouse/family/friends”). Responses ranged from 1 (“Never”) to 4 (“Always”).

#### Work Engagement

Work engagement was measured by the French version of the work engagement scale validated by Schaufeli et al. (2002, 2006). This instrument consists of 17 items that assess three dimensions of work engagement. Vigor is evaluated through six items (e.g., “At my job, I feel strong and vigorous”); dedication through five items (e.g., “To me, my job is challenging”); absorption through six items (e.g., “When I am working, I forget everything else around me”). Each item is rated on a seven-point scale ranging from “Never” to “Always.”

#### Problematic Uses of Internet

In order to assess executives’ relationship to the Internet and to determine the extent to which it may be problematic, we used the Internet Addiction Test (Young, 1998) in its validated version in French (Khazaal et al., 2008). Young borrowed the diagnostic criteria used for pathological gambling to define Internet addiction, considering that it is an impulse-control disorder. Young’s preference in describing these disorders as problematic rather than as addiction in the strict sense confers with other researchers.

The IAT is composed of 20 items that assess the degree of severity of the negative consequences resulting from excessive Internet use (e.g., “How often do you find that you stay online longer than you intended?”; “How often do you check your e-mail before something else that you need to do?”). The scale evaluates the frequency of occurrence of individuals’ behaviors, attitudes, and feelings as to their Internet use, as well as the negative consequences of these uses (on a five-point frequency scale from “Never” to “Always”).

### Analysis Strategy

Statistical analyses were run using SPSS and AMOS 21 software. We primarily used confirmatory factor analyses in order to validate the structure of our measurement tools. Several fit indices were used to assess each measurement scale: CMIN/ddl < 3 (Kline, 1998), CFI ≥ 0.95 (Hu and Bentler, 1999), GFI ≥ 0.95 (Schermelleh-Engel et al., 2003), AGFI ≥ 0.90 (Schermelleh-Engel et al., 2003), and RMSEA < 0.06 (Hu and Bentler, 1999). In addition, reliability analyses were carried out (i.e., Cronbach’s alpha coefficient). Finally, Pearson correlations were tested in order to examine the relationships among outcome variables.

#### Phase 1. Determine Categories of Internet Uses for Professional Purposes

With the goal of identifying typologies or profiles of daily Internet uses for professional purposes, a Hierarchical Cluster Analysis (HCA) was applied. HCA is a method used to identify groups of individuals that are more similar to each other across a number of observed variables, but less similar to individuals in different groups (Mooi and Sarstedt, 2011). This is an exploratory technique that consists of a number of consecutive steps, from which the most reliable cluster solution is generated. In hierarchical methods, the individuals under study are classified in groups at different stages, producing dendrograms that present the individuals and their respective points of junction or division for the groups formed in each stage.

As a measure of dissimilarity between groups, the squared Euclidean distance between each pair of observations was performed. As a procedure to group similar objects, Ward’s hierarchical clustering method was applied.

A discriminant analysis (DA) was used in conjunction with the HCA to validate the employed grouping methodology and to resolve classification problems and subsequent prediction of individuals under observation (Kinnear and Gray, 2012). The DA indicated that a four-cluster solution was the most appropriate (93.8% of the original observations are correctly classified).

Finally, chi–square tests of Pearson were performed to describe the characteristics and differences among the groups or clusters (Kinnear and Gray, 2012).

#### Phase 2. Identify the Links Between Situational and Socio-Demographic Characteristics and the Modalities of Internet Uses for Professional Purposes

A multinomial logistic regression was carried out to examine the association between situational and socio-demographic characteristics (i.e., personal Internet uses, technological equipment, work-related and individual characteristics) and executives’ Internet uses for work (Kinnear and Gray, 2012).

#### Phase 3. Apprehend the Way in Which the Modalities of Internet Uses for Professional Purposes Affect Work Engagement, Work–Non-work Interference, and the Relationship to the Internet

Finally, one-way analyses of covariance (ANCOVAs) were conducted to investigate the links between daily Internet uses for work clusters and perceived work–home interaction, work engagement, and the relationship to the Internet (Dancey and Reidy, 2011; Kinnear and Gray, 2012). Socio-professional and socio-demographic characteristics were included as covariates insofar as they were significantly related to the outcome variables. Hence, linear regressions and ANOVAs were performed beforehand.

## Results

### Psychometric Characteristics of Scales and Correlation Analysis

As shown in Table 2, the results obtained from confirmatory factorial analysis validated the following: the two-dimensional structure of the Work–Home Interaction scale (Wagena and Geurts, 2000), the three-dimensional structure of the Work engagement scale (Schaufeli and Bakker, 2003), and the one-dimensional structure of Internet Addiction Test (Young, 1998). However, to achieve a result that meets the criteria initially established, we had to remove three items from the Work–Home Interaction scale, eight items from the Work engagement scale, and four items from the IAT. The final structure of work–home interaction consists of 10 items (six items assess negative WNWI and four items positive WNWI). The final structure of work engagement consists of three items in each of the three sub-dimensions (vigor, dedication, absorption).

**TABLE 2:** Adjustment of the initial structure and the structure finally adopted to measure work–non-work interference(WNWI), work engagement, and problematic relationship to the Internet.

		**CMIN/ddl**	**CFI**	**GFI**	**AGFI**	**RMSEA**
W-NW interference	IS	3.529	0.92	0.93	0.91	0.07
	FS^a^	2.695	0.96	0.96	0.94	0.05
Work engagement	IS	5.460	0.88	0.84	0.80	0.09
	FS^b^	2.899	0.97	0.97	0.94	0.05
IAT	IS	3.653	0.88	0.88	0.85	0.07
	FS^c^	2.901	0.95	0.95	0.92	0.05

*IS, initial structure; FS, final structure; ^*a*^10 items; ^*b*^9 items, ^*c*^16 items.*

Table 3 presents the means, standard deviations, and reliabilities of the each scale/subscale. Although the alpha coefficients differed, ranging from 0.70 to 0.92, the six measures reached acceptable, even good levels of internal consistency.

**TABLE 3:** Means, standard deviations, Cronbach’s alpha, and correlations among outcome variables.

	**M (σ)**	**1**	**2**	**3**	**4**	**5**	**6**
1. Negative W-NW	13.86 (4.95)	(0.85)					
2. Positive W-NW	9.57 (3.26)	0.283^∗∗^	(0.74)				
3. Vigor	14.75 (3.42)	–0.071	0.023	(0.71)			
4. Dedication	15.44 (3.54)	–0.124^∗∗^	0.101^∗^	0.579^∗∗^	(0.83)		
5. Absorption	12.74 (3.47)	0.218^∗∗^	0.168^∗∗^	0.465^∗∗^	0.493^∗∗^	(0.70)	
6. Problematic Internet Uses	36.02 (13.68)	0.355^∗∗^	0.274^∗∗^	–0.339^∗∗^	–0.272^∗∗^	–0.033	(0.92)

*^∗^*p* < 0.05; ^∗∗^*p* < 0.01.*

According to Table 3, correlations between the two subdimensions of WNWI and between the three subdimensions of work engagement were significant, positive, and rather moderate. The results showed that both negative WNWI and positive WNWI were positively associated with higher levels of problematic Internet uses and absorption. Even though the correlations were weak, statistically significant associations between WNWI and dedication were also observed. Negative WNWI did co-vary with less dedication while a positive correlation was found between positive WNWI and this subdimension of work engagement. However, WNWI was not associated with vigor. In addition, it can be observed in Table 3 that vigor and dedication negatively relate to problematic Internet use. It is interesting to note that the correlation between absorption and problematic Internet uses is also negative but non-significant.

### Descriptive Statistics Associated With Work-Related Internet Uses

Regarding Internet use for work, as indicated in Table 4, the executives surveyed had rather moderate use when we compare their uses during a workday (around 4.16 h on average) to the uses during typical non-work periods (around 1 to 1.5 h on average and per day during days off or vacation). Even though the workplace was considered the preferred location for work *via* the Internet during a typical workday, the work-related uses at home were around 1 h per day on average, whatever the period being considered. Internet uses for professional purposes while traveling on public transport were also regular but less intensive (only a few minutes a day).

**TABLE 4:** Descriptive statistics of daily work-related Internet uses (IUPP).

	**M (σ)**	**Min–Max**
Internet uses for professional purposes (IUPP)–Workday	4.16 (2.53)	0.25–11.00
IUPP—Workday—At work	3.40 (2.28)	0.25–9.75
IUPP—Workday—At home	0.57 (0.80)	0.00–5.00
IUPP—Workday—In transports	0.19 (0.38)	0.00–3.50
Internet uses for professional purposes (IUPP)—Day off	1.42 (1.44)	0.00–10.00
IUPP—Day off—At home	1.12 (1.14)	0.00–8.00
IUPP—Day off—In transports	0.13 (0.35)	0.00–2.00
Internet uses for professional purposes (IUPP)—Day of leave	1.10 (1.32)	0.00–8.00
IUPP—Day of leave—At home	0.95 (1.14)	0.00–6.00
IUPP—Day of leave—In transports	0.10 (0.28)	0.00–2.00

With respect to technologies used, almost two in three respondents (64%) had a desktop computer for work, 39.9% a smartphone, 36.3% a laptop computer, and 5% a tablet provided by the employer. As illustrated in Table 1, the vast majority of executives (90%) used at least one device provided by their company (mainly desktop computer, laptop, or smartphone).

As for their relationship to the Internet for work, participants did not seem to have developed excessive tendencies (see Table 3). If we look at the numbers of respondents and the criteria set by Young (1998), 68% of respondents had control over their usage of the Internet (scores <40), while the remaining 32% had uses that create frequent or significant problems.

### Typology of Daily Internet Uses for Professional Purposes

**FIGURE 1:**
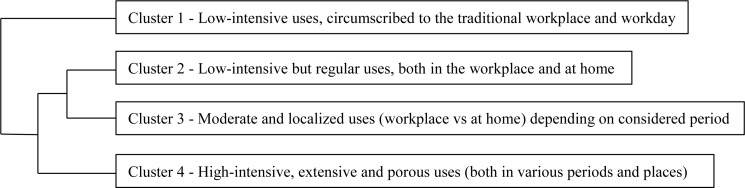
Typology of daily Internet uses for professional purposes [dendrogram from the Hierarchical Cluster Analysis (HCA) and the discriminant analysis (DA)].

Four distinct patterns of daily work-related Internet uses were identified (see Table 5 and Figure 1).

**TABLE 5:** Overrepresentation^a^ of the characteristics of work-related internet uses in each cluster.

	**Cluster 1**	**Cluster 2**	**Cluster 3**	**Cluster 4**	**Chi-square (df)**
Workday	0.25–2 h	0.25–2 h	2.25–4.75 h	5–11 h	χ^2^ (6) = 123,828^∗∗^
Workday—At work	0–2 h	0–2 h	2.25–4 h	4.25–9.75 h	χ^2^ (6) = 58,671^∗∗^
Workday—At home	0 h	0.25–5 h	0 h	0.25–5 h	χ^2^ (3) = 189,935^∗∗^
Workday—In transports	0 h	nd	nd	0.25–3.5 h	χ^2^ (3) = 42,878^∗∗^
Day off	0 h	0.25–1.75 h	2–10 h	2–10 h	χ^2^ (6) = 707,883^∗∗^
Day off—At home	0 h	0.25–1 h	0.25–1 h	1.25–8 h	χ^2^ (6) = 508,962^∗∗^
Day off—In Transports	0 h	nd	nd	0.25–2 h	χ^2^ (3) = 36,989^∗∗^
Day of leave	0 h	0.25–0.75 h	0.25–0.75 h	1–8 h	χ^2^ (6) = 627,913^∗∗^
Day of leave—At home	0 h	0.25–1 h	0.25–1 h	1.25–6 h	χ^2^ (6) = 540,614^∗∗^
Day of leave—In transports	0 h	nd	nd	0.25–2 h	χ^2^ (3) = 33,649^∗∗^

*^*a*^*Adjusted Residual* >*1.96;*^∗^*p* < 0.05; ^∗∗^*p* < 0.01; nd, non-discriminant.*

Cluster 1 (*N* = 124) was made up of executives who had low-intensity uses (less than 2 h per day), exclusively realized during working days and at the workplace. Therefore, they didn’t use the Internet for business purposes outside typical working hours and places.

Cluster 2 (*N* = 125) was composed of individuals with low-intensity (less than 2 h per day) but regular uses (effective whatever the period considered), both at work and at home.

Cluster 3 members (*N* = 122) reported moderate (between 2 and 10 h per day during a working day or a day off, but less than 1 h on a day’s leave) and very localized uses (in the workplace during working days and at home during non-working days). In other words, they used the Internet for work outside working hours exclusively during non-working days and from home.

Finally, executives who fell into Cluster 4 (*N* = 131) stated the most intensive uses whatever the period being considered (going up to 11 h per day). They used Internet for work at the office, at home, as well as while traveling on public transport.

### Association Between Situational and Socio-Demographic Characteristics and Internet Uses for Work

Logistic regression established that, all other things being equal, daily Internet uses for personal reasons and the number of technological devices provided by the employers, were significantly related to the uses of the Internet for work (see Table 6).

**TABLE 6:** Multinomial logistic regression: association between situational and socio-demographic characteristics and work-related Internet use categories.

		**Chi^2^ Wald (df = 1)**				**Exp(B)**			
		**C1**	**C2**	**C3**	**C4**	**C1**	**C2**	**C3**	**C4**
Personal internet use—workday	ref.C1	–	0.291	6.747^∗∗^	10.788^∗∗^	–	1.124	1.656	1.894
	ref.C2	0.291	–	4.268^∗^	7.687^∗∗^	0.889	–	1.473	1.685
	ref.C3	6.747^∗∗^	4.268^∗^	–	0.995	0.604	0.679	–	1.143
	ref.C4	10.788^∗∗^	7.687^∗∗^	0.995	–	0.528	0.594	0.875	–
Personal internet use—day off	ref.C1	–	8.089^∗∗^	1.573	2.293	–	0.593	0.801	0.766
	ref.C2	8.089^∗∗^	–	2.903	1.977	1.686	–	1.361	1.292
	ref.C3	1.573	2.903	–	0.100	1.239	0.735	–	0.949
	ref.C4	2.293	1.977	0.100	–	1.305	0.774	1.053	–
Personal internet use—day of leave	ref.C1	–	0.945	0.295	14.821^∗∗^	–	1.198	1.100	1.912
	ref.C2	0.945	–	0.223	7.651^∗∗^	0.835	–	0.918	1.596
	ref.C3	0.295	0.223	–	12.621^∗∗^	0.909	1.089	–	1.738
	ref.C4	14.821^∗∗^	7.651^∗∗^	12.621^∗∗^	–	0.523	0.626	0.575	–
Portable connecting tools-PE	ref.C1	–	10.627^∗∗^	26.703^∗∗^	33.555^∗∗^	–	3.085	5.931	8.156
	ref.C2	26.703^∗∗^	4.664^∗^	–	1.097	0.169	–	1.923	2.644
	ref.C3	33.555^∗∗^	9.330^∗∗^	1.097	–	0.123	0.520	–	1.375
	ref.C4	10.627^∗∗^	–	4.664^∗^	9.330^∗∗^	0.324	0.378	0.727	–
Age	ref.C1	–	0.688	0.055	0.250	–	0.988	0.996	1.008
	ref.C2	0.688	–	0.323	1.607	1.012	–	1.009	1.020
	ref.C3	0.055	0.323	–	0.533	1.004	0.991	–	1.012
	ref.C4	0.250	1.607	0.533	–	0.992	0.980	0.988	–
Gender	ref.C1	–	0.387	0.071	0.002	–	1.183	1.077	1.014
	ref.C2	0.387	–	0.121	0.294	0.845	–	0.910	0.857
	ref.C3	0.071	0.121	–	0.046	0.929	1.099	–	0.942
	ref.C4	0.002	0.294	0.046	–	0.986	1.167	1.062	–
Children	ref.C1	–	0.108	1.497	0.917	–	–	1.130	0.832
	ref.C2	0.108	–	0.856	1.647	0.958	1.044	1.179	0.868
	ref.C3	1.497	0.856	–	4.638^∗^	0.848	0.885	–	0.736
	ref.C4	0.917	1.647	4.638^∗^	–	1.151	1.202	1.358	–
Household composition	ref.C1	–	0.705	1.468	0.014	–	0.878	0.826	0.982
	ref.C2	0.705	–	0.146	0.521	1.139	–	0.941	1.119
	ref.C3	1.468	0.146	–	1.277	1.211	1.063	–	1.189
	ref.C4	0.014	0.521	1.277	–	1.018	0.894	0.841	–
Socio-professional category (SPC)	ref.C1	–	1.474	0.014	0.394	–	0.711	0.967	0.825
	ref.C2	1.474	–	1.184	0.245	1.407	–	1.360	1.616
	ref.C3	0.014	1.184	–	0.290	1.034	0.735	–	0.853
	ref.C4	0.394	0.245	0.290	–	1.212	0.862	1.172	–
Work sector	ref.C1	–	1.384	0.140	1.465	–	1.410	0.890	1.481
	ref.C2	1.384	–	2.293	0.024	0.709	–	0.631	1.050
	ref.C3	0.140	2.293	–	2.481	1.124	1.585	–	1.664
	ref.C4	1.465	0.024	2.481	–	0.675	0.953	0.601	–

*–2LogL, 959,73; *R*^2^ Cox & Snell, 0.28; *R*^2^ Nagelkerke, 0.29; ^∗^*p* < 0.05. ^∗∗^*p* < 0.01.*

The respective effects of the variables (continuous or nominal) are evaluated with regard to a category (i.e., cluster) serving as a reference in the equation. An odds ratio [i.e., Exp(B)] greater than 1 indicates that when the value of the explanatory variable increases by one, the probability of belonging to the category of use considered increases (and this with regards to the category of reference). Conversely, an odds ratio less than 1 indicates that when the value of the explanatory variable increases by one, the probability of belonging to the category of use considered decreases (and this with regard to the category of reference).

Results indicated that the more executives used the Internet for personal purposes, whether during workdays or during their vacation, the more they had intensive uses of the Internet for work (Clusters 3 and 4). Conversely, they were less likely to had work-related low-intensity uses of the Internet (Clusters 1 and 2). However, personal uses during days off were positively associated with low-intensity uses of the Internet for work during working hours (Cluster 1 vs. Cluster 2).

In addition, all else being equal, the more technological devices provided by the employers, the more executives used the Internet for business purposes, both during working hours at the workplace and outside of the standard workday/workplace (Cluster 4 and 3 vs. Clusters 2 and 1).

Furthermore, there were no significant differences between the four categories of daily work-related uses according to individual characteristics and to the family or socio-professional situation (age and gender, number of children and household composition, SPC and work sector). Nevertheless, it is interesting to note that the more respondents had children, the less likely they were to have intensive, extensive, and porous uses of the Internet for professional purposes (Cluster 4 vs. Cluster 3).

### Association Between Internet Uses for Work and Work–Non-work Interference, Work Engagement, and Relationship to the Internet

#### Preliminary Results

The decision as to which covariates to include in the final analyses was specified on the basis of data from linear regressions and ANOVAs.

As can be seen in Table 3, the results showed significant relationships between some socio-professional and socio-demographic characteristics and the three explained variables.

Overall, high-intensity uses of the Internet for personal purposes fostered negative WNWI and problematic Internet use, whereas it diminished work engagement. However, these relationships were dependent on periods considered (see Table 7).

**TABLE 7:** Association between situational and socio-demographic characteristics and the perceived work–home interaction.

	**→ Negative W-NW**			**→ Positive W-NW**		
**Linear regression**	***F* (df = 1)**	**β**	***R*^2^**	***F* (df)**	**β**	***R*^2^**
PIU—Workday	4.292	0.092^∗^	0.007	1.852	0.061	0.002
PIU—Day off	1.812	–0.060	0.002	0.001	–0.001	0.000
PIU—Day of leave	0.045	0.009	0.000	0.013	–0.005	0.000
PCT-PE	17.975	0.186^∗∗^	0.033	9.178	0.134^∗∗^	0.016
Age	1.554	–0.056	0.001	0.775	–0.039	0.000
Children	0.004	–0.003	0.000	1.182	–0.049	0.000

**ANOVA**		***F* (df)**		**η^2^**	***F* (df)**	**η^2^**

Gender		1.000 (1)		0.002	2.261 (1)	0.005
Household composition		0.373 (4)		0.000	1.000 (4)	0.002
Socio-professional category (SPC)		0.063 (1)		0.000	0.001 (1)	0.000
Work sector		0.104 (1)		0.000	0.250 (1)	0.000

**R*^2^, adjusted *R*-squared; η^2^, partial Eta-squared; ^∗^*p* < 0.05; ^∗∗^*p* < 0.01.*

Moreover, the more technological devices provided by the employer, the more work was perceived as having negative as well as positive impacts on personal life, and the more executives were engaged in Internet behaviors that tended to be excessive (see Tables 7, 9).

Furthermore, the older respondents were, the more they were engaged at work and the less they had problematic Internet use. The results also indicated that women were significantly less absorbed by their work than men (*M* = 12.40; σ = 3.71 vs. *M* = 13.05; σ = 3.22), that executive officers, CEOs, or senior executives were more dedicated to their work than middle managers (*M* = 15.92; σ = 3.14 vs. *M* = 14.78; σ = 3.75), and that executives with a high number of children reported significantly lower problematic use of the Internet (see Tables 8, 9). These results confirm and complement previous researches which showed that men scored significantly higher than women on absorption and that managers exhibited one of the highest scores in all dimensions compared to other occupational groups (Schaufeli and Bakker, 2003). Moreover, they suggest that family caregiver responsibilities limit time and resource allotted to Internet uses.

**TABLE 8:** Association between situational and socio-demographic characteristics and work engagement.

	**→Vigor**			**→Dedication**			**→Absorption**		
**Linear regression**	***F* (df = 1)**	**β**	***R*^2^**	***F* (df = 1)**	**β**	***R*^2^**	***F* (df = 1)**	**β**	***R*^2^**
PIU—Workday	7.567	–0.122^∗∗^	0.013	7.657	–0.123^∗∗^	0.013	0.646	–0.036	0.001
PIU—Day off	4.178	−0.091^∗^	0.006	1.136	–0.048	0.000	4.173^∗^	−0.091^∗^	0.006
PIU—Day of leave	4.064	−0.090^∗^	0.006	0.270	–0.023	0.001	1.167	–0.048	0.000
PCT-PE	1.084	–0.047	0.000	0.019	–0.006	0.000	1.373	0.052	0.001
Age	9.969	0.140^∗∗^	0.018	4.092	0.090^∗^	0.006	9.454	0.136^∗∗^	0.017
Children	1.204	0.049	0.000	2.246	0.067	0.002	0.830	0.041	0.000

**ANOVA**	***F* (df)**		**η^2^**	***F* (df)**		**η^2^**	***F* (df)**		**η^2^**

Gender	0.069 (1)		0.000	2.743 (1)		0.006	4.459 ^∗^ (1)		0.009
Household composition	1.340 (4)		0.012	0.782 (4)		0.007	2.203 (4)		0.002
SPC	1.919 (1)		0.004	13.521^∗∗^ (1)		0.026	3.480 (1)		0.008
Work sector	1.595 (1)		0.004	0.786 (1)		0.002	0.014 (1)		0.000

**R*^2^, adjusted *R*-squared; η^2^, partial Eta-squared; ^∗^*p* < 0.05,; ^∗∗^*p* < 0.01.*

**TABLE 9:** Association between situational and socio-demographic characteristics and relationship to the Internet.

	**→Problematic Internet Uses**		
**Linear regression**	***F* (df = 1)**	**β**	***R*^2^**
PIU—Workday	58.054	0.323^∗∗^	0.102
PIU—Day off	50.939	0.304^∗∗^	0.091
PIU—Day of leave	47.682	0.295^∗∗^	0.085
PCT-PE	4.720	0.097^∗^	0.007
Age	24.300	–0.215^∗∗^	0.044
Children	7.650	–0.123^∗∗^	0.013

**ANOVA**	***F* (df)**		**η^2^**

Gender	1.577 (1)		0.004
Household composition	2.373 (4)		0.005
SPC	0.349 (1)		0.000
Work sector	1.058 (1)		0.002

**R*^2^, adjusted *R*-squared; η^2^, partial Eta-squared; ^∗^*p* < 0.05; ^∗∗^*p* < 0.01.*

#### Work-Related Internet Uses and Work–Non-work Interference, Work Engagement, and Relationship to the Internet

The results that emerged from our analyses showed that using the Internet for work was significantly related to the outcome variables investigated (see Table 10).

**TABLE 10:** Association between categories of work-related internet uses and work–non-work interaction, work engagement, and relationship to the internet.

**ANCOVA**	**Covariates**	***F* (df)**	**η^2^**
Internet uses for professional purposes → negative WNWI	PIU—Workday PCT-PE	12.165^∗∗^ (3)	0.068
Internet uses for professional purposes → positive WNWI	PCT-PE	8.576^∗∗^ (3)	0.049
Internet uses for professional purposes → vigor	PIU—Workday PIU—Day off PIU—Day of leave Age	4.998^∗∗^ (3)	0.029
Internet uses for professional purposes → dedication	PIU—Workday Age SPC	5.994^∗∗^ (3)	0.035
Internet uses for professional purposes → absorption	PIU—Day off Age Gender	2.305 (3)	0.014
Internet uses for professional purposes → problematic Internet use	PIU—Workday PIU—Day off PIU—Day of leave PCT-PE Age Children	23.776^∗∗^	0.125

*η^2^, partial Eta-squared; ^∗^*p* < 0.05; ^∗∗^*p* < 0.01.*

After controlling for the intensity of personal uses of the Internet during working days and the number of technological devices provided by the employer, executives with low-intensity uses, circumscribed to the traditional workplace and workday, had a statistically significant lower mean score on perception of negative work–home interference (Tukey’s HSD *p* < 0.01; *M*_C__1_ = 11.69; σ = 3.73). Similarly, more intensive and porous Internet uses for work were associated with the perception of work–family conflict (*M*_C__2_ = 14.10; σ = 4.77 - *M*_C__3_ = 14.45; σ = 3.41 - *M*_C__4_ = 15.11; σ = 5.01). In addition, the results showed that the most intense, extensive, and porous uses of Internet for professional purposes were significantly related to higher perception of positive impact of work on personal life (Tukey’s HSD *p* < 0.01; *M*_C__4_ = 14.02; σ = 3.25). The means of the least intensive uses profiles were significantly lower (*M*_C__1_ = 12.06; σ = 0.31; *M*_C__2_ = 12.76; σ = 3.55). Cluster 3′s mean did not differ significantly from others (*M*_C__3_ = 13.77; σ = 0.32). In other words, Internet use for extended work favored the permeability between work life and personal life (perceived negative but also positive impacts of work). Hence, our first hypotheses (i.e., H1a and H1b) are fully confirmed.

Examination of ANCOVA’s results showed that executives’ work engagement scores, adjusted for age and personal uses differences, were different across work-related Internet use profiles. According to pairwise comparison tests, using the Internet for work with low intensity favors vigor (Tukey’s HSD *p* < 0.05: *M*_C__1_ = 14.98; σ = 3.53 - *M*_C__2_ = 15.48; σ = 2.75). On the contrary, executives with the most intense, extensive, and porous uses of Internet for professional purposes scored significantly low on vigor (*M*_C__4_ = 13.89; σ = 3.79). Cluster 3′s mean did not differ significantly from others (*M*_C__3_ = 14.70; σ = 3.35). Furthermore, executives with low-intensity but regular uses of Internet for work were significantly more likely to have a higher level of dedication than executives with work-related uses outside the usual workplace and workday (Tukey’s HSD *p* < 0.01: *M*_C__2_ = 16.52; σ = 3.45 vs. *M*_C__3_ = 15.20; σ = 3.69 - *M*_C__4_ = 14.82; σ = 2.88). However, the ANCOVA test of the effect of Internet uses for professional purposes on absorption was not significant. Our second hypothesis (H2) was only partially supported.

Finally, using the Internet for work seemed to encourage Internet attitudes and behaviors that tended to be excessive. Indeed, executives with the most intense uses of Internet for professional purposes had a statistically significant higher mean scores on IAT than executives with less intensive uses (Tukey’s HSD *p* < 0.01: *M*_C__4_ = 33.98; σ = 11.84 – *M*_C__3_ = 30.91; σ = 11.36 vs. *M*_C__2_ = 25.26; σ = 8.66 – *M*_C__1_ = 24.89; σ = 8.71). ANCOVA’s results thus suggested that the more managers worked *via* the Internet, the more they showed problematic Internet use. Therefore, our last hypothesis (H3) was confirmed.

## Discussion

### Theoretical Implications

According to what some research in the field may lead us to predict, the four cluster profiles identified revealed that Internet use at work is linked to the prolonging of work *via* technology (Rey and Sitnikoff, 2004; Boswell and Olson-Buchanan, 2007; Fenner and Renn, 2010; Senarathne Tennakoon et al., 2013; Thomas, 2014). In other words, using the Internet at work encourages supplemental work practices (Clusters 2, 3, and 4). Nonetheless, these results enabled us to deepen and refine our understanding of the link between work-related Internet uses in and outside the traditional workplace and workday. Indeed, the specific segmentation strategy adopted by Cluster 3 members suggests that this relationship is not perfectly linear. While they consolidate and complement existing researches, they need to be supplemented by additional studies designed to characterize in detail the multiple kinds of ICT uses for work.

Moreover, study findings are consistent with those from previous researches about the positive association between work done through technologies outside usual working hours and workplaces and the perception of work–family conflict (Boswell and Olson-Buchanan, 2007; Fenner and Renn, 2010; Leonardi et al., 2010; Ayyagari et al., 2011; Matusik and Mickel, 2011; Park and Jex, 2011; Thomas, 2014; Wright et al., 2014; Ferguson et al., 2016; Leung and Zhang, 2017; Carlson et al., 2018). Similarly, a segmentation strategy of work-related use of the Internet (Cluster 1) reduces the perception of work–life conflict. But if we consider together the dimensions related to work–home interaction, our results also concur with studies showing that supplemental work helps blur the boundaries between work and personal life (Isaac et al., 2007; Park and Jex, 2011; Tabassum and Rahman, 2013; Thomas, 2014; de Wet and Koekemoer, 2016). Here, we noted a significant porosity between work and non-work (work was perceived as having negative as well as positive impacts on personal life) related to the use of the Internet for work outside traditional workplaces and hours. As the literature suggests, ICT use can facilitate extending work into the non-work domain, turning the home into a workplace while at the same time promoting the emergence and management of personal concerns at work (Rey and Sitnikoff, 2004; Chesley, 2014; de Wet and Koekemoer, 2016). Indeed, managers often state that they are just as inclined to bring personal affairs into the office, justifying these practices through a rationale of compensation: if the supplementary hours of work encroach on personal life, then the reverse is also legitimate (Le Douarin, 2007). The results of our first analyses, showing that the intensity of personal use during a working day is positively related to professional use outside places and time periods commonly dedicated to work, reinforce this latter interpretation. These complementary findings may explain the observed effects on the perception of a positive impact of work on personal life that a few studies have suggested (Batt and Valcour, 2003; Wajcman et al., 2008; Land and Taylor, 2010; Chesley, 2014; Ninaus et al., 2015). Here, we can see the importance of understanding both the positive and the negative aspects of the impact of work on personal life. First, studies show that these dimensions of work/non-work are not necessarily the opposites (Geurts et al., 2005). Second, if one wishes to fully understand how the uses of Internet affect the work–life relationship, we cannot limit our research to looking only at the conflict between these two areas, the use of technology being also associated with work–family enrichment (Derks et al., 2016; Ghislieri et al., 2017).

Furthermore, the results showed that work-related Internet uses were significantly related to work engagement, thereby complementing studies in the field that rarely focus directly on these aspects. However, the way in which using the Internet for work affects work engagement is more subtle and targeted than previous research would seem to predict. Firstly, executives with the most extensive, porous, and high-intensive Internet uses for work are less likely to be vigorous and devoted at work (Cluster 4). This result confirms those put forward in the literature review, which points out that the uses of ICTs for work diminished organizational commitment and work engagement (Boswell and Olson-Buchanan, 2007; Salanova and Llorens, 2009; Ferguson et al., 2016; Ter Hoeven et al., 2016). As suggested by previous studies, using ICT for work after hours reflects a significant investment in the working world, and even work overload (Boswell and Olson-Buchanan, 2007; Fenner and Renn, 2010; Senarathne Tennakoon et al., 2013). This result may be interpreted as a sign of executives’ frustration even burnout. This interpretation is borne out by researches on this subject, which showed that job demands reduce engagement and that engagement and burnout are negatively correlated (Schaufeli et al., 2002, 2006; Bakker et al., 2006). This assumption remains nevertheless to be confirmed by future studies. Secondly, the value of multidimensional approaches for understanding work engagement also emerged from this study. The results clearly show that professional uses of ICTs and the relationship to the Internet did not act in the same way and on the same dimensions of engagement. While executives with low-intensity uses circumscribed to the traditional workplace and workday are the most vigorous (Cluster 1), executives with low-intensity uses both at work and at home are the most dedicated to their work (Cluster 2). By limiting their use of Internet, they would implement set times and/or days during which they would avoid using their ICT devices. Hence, executives appear to have created effective strategies for boundary management by actively restricting their work-related Internet uses both at work and at home, which may explain their vigor and dedication. However, it is interesting to note that work-related use of the Internet is not significantly associated with absorption, a sub-dimension that seems to function in a particular way.

Our findings also consolidate and supplement research on excessive Internet attitudes and behaviors. According to what has been advanced in previous studies, our results indicate that there is a significant and positive link between using the Internet for work and behavioral addiction to the Internet (Perlow, 2012; Salanova et al., 2013; Derks and Bakker, 2014; Wright et al., 2014; Li and Lin, 2019). When work-related Internet uses are low-intensive and remain circumscribed to the traditional workplace and workday, they are associated with the ability to control Internet usage. From the moment these uses are more intensive and go beyond this sphere, they can lead to problematic behaviors related to the Internet, which have considerable negative consequences (Widyanto and McMurran, 2004; Durand et al., 2008).

The use of ICTs for professional purposes, in and outside the traditional workplace and workday, seems to be a relevant variable for understanding the perceived impact of work on personal life, work engagement, and problematic Internet uses. In the light of our findings, it can be assumed that prolonging work *via* the Internet has rather deleterious effects. Nevertheless, while our results complement and refine existing researches, they need to be supplemented by studies encompassing all work uses of ICTs, both in and out of the office, and on their respective effects.

### Practical Implications

With the increasing digitalization of business processes and the mobilization of employees, mobile technologies have become indispensable tools. Companies massively provide employees with technological devices without establishing true policies and guidelines for regulating ICT uses, in order to ensure that employees’ time off and vacation time are respected as well as their personal and family life. One of the most important results of this study underlines that technological devices provided by the employers promote intensive uses of the Internet for business purposes, specifically outside of the standard workday and workplace (which in turn may have deleterious consequences). This result concurs with observations in this field and emphasizes the role of social norms, organizational demands, and culture that promote the extension of work outside the usual times and places *via* ICTs (Matusik and Mickel, 2011; Waller and Ragsdell, 2012; Senarathne Tennakoon et al., 2013; Thomas, 2014; Wright et al., 2014; de Wet and Koekemoer, 2016; Kossek, 2016). The study of Derks et al. (2015) highlights this phenomenon. They showed that for employees who perceive high availability expectations of their supervisor, smartphone use is strongly positively related to work than for employees without these expectations. In a complementary way, Fenner and Renn (2010) found that organization’s expectation and the social norms it conveys are interpreted by employees as pressure to use technologies to continue working at home after hours. Finally, Ninaus et al.’s (2015) study results showed that the provision of technological devices by the employer increases availability pressure, regardless of whether or not this is explicitly expected.

The ability to disconnect from work and to separate work and personal life is a real skill, involving rigorous organization and control of the spaces and times devoted to different activities (Vayre and Pignault, 2014). This skill is crucial, since workers increasingly have an obligation to produce results no matter what; work tasks are no longer organized within a given time frame or place, but by goals to be reached, meaning that workers are responsible for managing their work time (Rey and Sitnikoff, 2004). Today, questions about boundaries and work–life balance have led to discussions about the “right to disconnect,” whether during free time or the workday. This new right includes a right to be alone, to not be disturbed, being able to take time to step back and reflect, and not being obliged to respond immediately to a phone call or to an e-mail, for example. Since 2010, some large companies and groups, including some in France, have developed charters for appropriate Internet use, notably e-mail, during or outside working hours. However, these organizational policies and arrangements must be expanded and are still too scarce in French companies.

At the same time, prevention programs about Internet use are also starting to be developed and applied, but remain very rare. They involve informing employers and employees of the risks incurred by unregulated and uncontrolled use of work cell phones, e-mail, Internet, etc. However, unlike the United States, in France, the problematic uses of the Internet or even cyber-addiction are still rarely taken seriously: support organizations are insufficient and prevention is virtually non-existent. As the use of ICT and devices is widely valued and encouraged by society, it is easy to understand that cyber-addiction is more socially accepted compared to other addictions among professional/managerial circles. In addition, the various pressures from the work environment on employees may generate, or at least fuel, problematic or addictive attitudes and behavior toward digital technologies and the Internet (Salanova et al., 2013). Indeed, current organizational contexts advocate efficiency at all costs, permanent availability, and setting too high or unattainable goals, which are factors in developing addictive behaviors at work (Durand et al., 2008). Considering these contexts and the demands they convey, we may expect that behavioral addiction to technologies or at least excessive use of the Internet would even give a positive image of the employee (as is the case for workaholism).

It is therefore necessary to continue with and strengthen the reflections, guidelines, and practices already adopted in certain work organizations (and when this is not the case, initiating dialogue within companies). This research could provide valuable information on the promotion of employee health and quality of life in the concerned fields and may constitute a suitable basis for developing interventions and preventative measures on an organizational level, in controlling the negative outcomes of work-related ICTs’ uses. Observations from empirical work on this subject can inform discussions and decisions within work organizations and raise awareness among managers and supervisors about the adverse effects of ICT use for extended work on porosity between work and personal life, work engagement, and problematic Internet use. As seen in the literature review, these have widely recognized consequences on behavior and attitudes at work, on performance, on workers’ well-being and health, and on organizations’ efficiency and productivity.

### Limitations and Future Avenues of Research

This research offers a starting point for investigating the effects of work use of communication and Internet technology on different aspects of work and the relation between work and personal life. Although original and highly informative, the study does have some limitations. First, the research design is cross-sectional, while longitudinal designs allow for stronger conclusions concerning possible causal relationships among variables. Second, since it is based on a self-reported questionnaire, it can be assumed that social desirability biases are involved in responses on the intensity of Internet use for work as well as for exhibiting addictive uses. It is likely that the uses declared are lower than actual use. Third, the study focuses on Internet uses and thus does not take into account other ICTs uses. Therefore, there may be differentiated effects that depend on the devices or technology used. Fourth, the focus has solely been on work–home interference, work engagement, and relationship to the Internet, neglecting the study of other dimensions of work, health, and quality of life of workers, which are important. Finally, the study is based on a somewhat small sample focused on managers and professionals.

Given the contributions and limitations of this study, it is important to conduct future empirical studies to examine the effects of work-related ICT uses for work in greater detail to better understand how and to what extent the working world is encroaching on personal life, and the role of ICTs in defining the work–life relationship. To do so, it would be beneficial to complement this study with data collection methods to counter self-reporting bias and overcome weaknesses of the cross-sectional design, such as complementary measurements of ICT use (software to track subject’s activity, surveying at different times of the day/week *via* a diary study, etc.). We also believe that participants’ significant others should be interviewed, since respondents tend to underestimate and downplay the perception of conflict, insofar as extended work is likely to be a source of professional gratification or reward (Boswell and Olson-Buchanan, 2007).

In addition, it could be appropriate, in the context of future research, to explore the potential links between professional Internet uses, the relationship to Internet, and the relationship to work. It can be assumed that there is a positive link between these three dimensions in so far as the research already conducted indicate for example that work addiction presents negative consequences on the work–family conflict (Taris et al., 2005; Bakker et al., 2009) and that working compulsively and the three dimensions of engagement are negatively correlated (Schaufeli et al., 2008).

Furthermore, additional studies should be designed to characterize in detail the multiple kinds of ICT uses for work (the technologies used, the places and times associated with uses, applications or software used, etc.) to identify any differentiated effects. Doing so would enable us to determine which uses may have harmful consequences or, on the contrary, effects that are beneficial for accomplishing work, both for the employee and the organization.

The Job Demands Resources model may be used as an integrative conceptual framework for future investigations in this area (JD-R Model; Bakker and Demerouti, 2017). Indeed, to grasp the experience of work and understand the interplay between constellations of job demands/resources and health-impairment/motivational processes, it would be useful to fully examine the different ways in which ICTs can be used for work.

## Data Availability Statement

The datasets for this manuscript are not publicly available due to ethical and confidentiality considerations. Requests to access the datasets should be directed to EV, emilie.vayre@univ-lyon2.fr.

## Ethics Statement

The studies involving human participants were reviewed and approved by Institutional Ethical Committee (Paris Nanterre University). The patients/participants provided their written informed consent to participate in this study.

## Author Contributions

Both authors listed have made substantial, direct, and intellectual contribution to the work and approved it for publication.

## Conflict of Interest

The authors declare that the research was conducted in the absence of any commercial or financial relationships that could be construed as a potential conflict of interest.

## Footnotes

1 Participants were recruited by the researchers.
